# Comparison of Survival Outcomes Among Women With Breast Cancer Across Different Health Systems in Veracruz, Mexico

**DOI:** 10.7759/cureus.104635

**Published:** 2026-03-03

**Authors:** Carlos Adrián Alarcón Rojas, María Teresa Alvarez Bañuelos, Jaime Morales-Romero, Raúl Enrique Guzmán García

**Affiliations:** 1 Epidemiology and Public Health, Faculty of Bioanalysis, Veracruzana University, Xalapa, MEX; 2 Public Health Institute, Veracruzana University, Xalapa, MEX; 3 Oncology, State Center for Diagnostic Evaluation of Breast Cancer, Xalapa, MEX

**Keywords:** breast cancer prognosis, health services access, kaplan-meier survivorship, risk factors‎, social determinant of health

## Abstract

In Mexico, breast cancer (BC) survival is influenced by stage at diagnosis, sociodemographic characteristics, and access to health services. This study aims to compare overall survival and associated sociodemographic and clinical characteristics among women with BC treated in two health systems in Veracruz, Mexico. Clinical records of women diagnosed between 2012 and 2021 were reviewed. A total of 500 medical records were initially screened (250 per institution); after applying inclusion and exclusion criteria, 464 patients were included in the final cohort. Statistical analyses included descriptive statistics, proportions, and mean difference tests. Survival probabilities were estimated using the Kaplan-Meier method, with group comparisons assessed by the log-rank test. Multivariate associations were evaluated using Cox proportional hazards regression adjusted for sociodemographic and clinical variables (age, clinical stage, education level, occupation, area of residence, lymph node involvement, molecular subtype, and health institution).

The mean age was 55 ± 12 years. Insured women were more frequently employed, had higher educational attainment, lived in urban areas, and were diagnosed at earlier clinical stages. In contrast, uninsured patients were more often homemakers, had basic education, lived in rural areas, and presented with advanced disease (p ≤ 0.05). Five-year Kaplan-Meier survival was 97.5% (n = 229) among insured patients compared with 83.1% (n = 204) among uninsured patients. In the adjusted Cox model, uninsured patients showed a higher risk of death (hazard ratio (HR) = 4.70; 95% confidence interval (CI): 1.66-13.52).

These findings indicate that health insurance status and institutional context are associated with differences in patient characteristics and survival. The results highlight the need to strengthen equity and standardization in care processes to improve early diagnosis and reduce BC mortality in Veracruz.

## Introduction

Over the past decade, breast cancer (BC) has become a major public health concern due to its increasing global incidence, associated with population aging, urbanization, sedentary lifestyles, and dietary changes [1]. Although the disease is more frequently diagnosed in high-income countries, most deaths occur in low- and middle-income settings, where advanced stages at diagnosis and delays in treatment initiation remain common [2-4].

In Mexico, BC represents a growing health burden, characterized by increasing mortality, frequent late-stage presentation, and reduced survival. Between 2012 and 2020, mortality rates continued to rise nationwide, with increases greater than 25% in some states [5,6]. However, evidence on the combined influence of sociodemographic, clinical, and tumor-related factors on survival across different healthcare settings remains limited.

Previous studies have identified clinical stage, tumor characteristics, and age at diagnosis as important predictors of survival [7]. Additionally, socioeconomic conditions and health insurance status have been associated with differences in stage at diagnosis and mortality, reflecting disparities in access to timely diagnosis and treatment [8,9]. In the Mexican context, health system delays have been documented, with treatment initiation occurring weeks or months after diagnosis, contributing to more advanced disease and poorer outcomes [4].

Given the structure of the Mexican health system and potential differences in patient characteristics and access to care, evaluating survival across health institutions may provide relevant information to identify inequities and inform improvements in healthcare delivery.

This study aimed to compare overall survival and associated clinical and sociodemographic characteristics among women with BC treated in two health systems in Veracruz: The Regional High Specialty Hospital of the Institute of Security and Social Services for State Workers (ISSSTE) and the State Health Services at the High Specialty Center “Dr. Rafael Lucio” and the State Cancer Center “Dr. Miguel Dorantes Mesa.”

## Materials and methods

Study design and setting

A retrospective cohort study was conducted using medical record review of women diagnosed with BC between January 2012 and December 2021. Patients were treated in two healthcare systems in Veracruz, Mexico: State Health Services (SESA: CAE and CECAN), which served the uninsured population, and the Regional High Specialty Hospital of the ISSSTE, which served insured patients.

Participants and data collection

Women aged ≥18 years with histopathologically confirmed BC and available follow-up information were included. The minimum sample size was estimated using a five-year survival proportion of 81.77%, a 95% confidence level, and 5% precision, resulting in 229 patients per system. To account for incomplete records, 250 files per system were reviewed; the final cohort included 464 patients (232 per institution).

Data were extracted using a standardized form and included sociodemographic characteristics (age, education, occupation, residence), clinical variables (menopausal status, comorbidities, body mass index, family history, tobacco and alcohol use), and tumor-related information (histology, grade, clinical stage, lymph node status, metastasis, and molecular subtype). Double data entry and verification were performed to minimize information errors.

Outcome and follow-up

Overall survival was defined as the time from diagnosis to death from any cause or last contact. Vital status was obtained from clinical follow-up records and death certificates when available. In cases without recent information, status was confirmed by telephone using contact data recorded in the file; when applicable, death was verified through verbal confirmation from relatives. No linkage with national mortality registries was performed.

Statistical analysis

Patient characteristics were summarized using descriptive statistics. Group comparisons were performed using chi-square tests and Student’s t-test or Mann-Whitney U test, as appropriate. Survival was estimated using Kaplan-Meier curves and compared with the log-rank test.

Multivariable Cox proportional hazards models were used to estimate hazard ratios (HRs) and 95% confidence intervals (CIs), adjusting for age, education, occupation, residence, clinical stage, lymph node involvement, molecular subtype, and healthcare system. Variables were selected based on epidemiological relevance and bivariate analysis. The proportional hazards assumption was evaluated using Schoenfeld residuals. Analyses were performed using R software (R Foundation for Statistical Computing, Vienna, Austria), with p ≤ 0.05 considered statistically significant.

Ethical considerations

The study was classified as minimal risk, and the requirement for written informed consent was waived due to its retrospective design. Survival verification through relatives involved verbal confirmation only. The protocol, including follow-up procedures and data verification, was approved by the ethics and research committees of all participating institutions and by the Institute of Public Health (CEI-ISP) of the Veracruzana University (CEI-ISP-UV-R16/2022). Data were anonymized before analysis.

## Results

The study included women treated in insured and uninsured health systems across two institutions. During the sampling process, 250 medical records were reviewed at each institution; 18 records (7.2%) did not meet the inclusion criteria. The final cohort comprised 464 patients, with 232 patients in each group. The overall mean age at diagnosis was 55 ± 12 years (range: 20-89 years).

Comparisons of sociodemographic and clinical characteristics between insured and uninsured patients showed statistically significant differences across several variables (Table 1). Strong associations, based on Cramér’s V, were observed between insurance status and occupation (χ^2^(1) = 175.14, p < 0.001, Cramér’s V = 0.61) and between insurance status and education level (χ^2^(1) = 133.86, p < 0.001, Cramér’s V = 0.54), indicating marked socioeconomic differences between groups.

**Table 1:** Sociodemographic and clinical characteristics of breast cancer patients according to health insurance status. χ^2^: chi-square statistic; df: degrees of freedom; Cramér’s V: effect size for chi-square tests; Phi coefficient was used for 2×2 contingency tables. Percentages are presented within each group.

Variable	Insured (n = 232), n (%)	Uninsured (n = 232), n (%)	χ^2^	df	p-value	Cramér’s V
Age						
<40 years	18 (7.7)	31 (13.4)	3.86	1	0.050	0.09
≥41 years	214 (92.3)	201 (86.6)				
Occupation						
Housewife	76 (32.8)	214 (92.2)	175.14	1	<0.001	0.61
Employed	156 (67.2)	18 (7.8)				
Education level						
Basic	73 (31.5)	196 (84.5)	133.86	1	<0.001	0.54
High school or higher	159 (68.5)	36 (15.5)				
Residence						
Rural	76 (32.8)	119 (51.3)	16.36	1	<0.001	0.19
Urban	156 (67.2)	113 (48.7)				
Parity						
Yes	183 (78.9)	209 (90.1)	11.10	1	0.001	0.16
No	49 (21.1)	23 (9.9)				
Histological grade						
Differentiated	22 (9.5)	48 (21.1)	11.50	1	0.001	0.16
Poorly differentiated	210 (90.5)	183 (78.9)				
Clinical stage						
Early	145 (62.5)	86 (37.1)	30.00	1	0.001	0.25
Advanced	87 (37.5)	146 (62.9)				
Body mass index						
Normal weight	49 (21.1)	49 (21.1)	7.49	3	0.058	0.13
Overweight	97 (41.8)	79 (34.1)				
Obesity	69 (29.8)	94 (40.5)				
Morbid obesity	17 (7.3)	10 (4.3)				
Lymph node involvement						
Yes	100 (43.1)	171 (73.7)	44.72	1	<0.001	0.31
No	132 (56.9)	61 (26.3)				
Molecular subtype						
Luminal A	150 (64.6)	133 (57.3)	3.25	3	0.355	0.08
Luminal B	33 (14.2)	35 (15.1)				
HER2	15 (6.5)	22 (9.5)				
Triple negative	34 (14.7)	42 (18.1)				

Moderate associations were identified for lymph node involvement (χ^2^(1) = 44.72, p < 0.001, Cramér’s V = 0.31) and clinical stage (χ^2^(1) = 30.00, p = 0.001, Cramér’s V = 0.25). Small but statistically significant associations were observed for place of residence (χ^2^(1) = 16.36, p < 0.001, Cramér’s V = 0.19), parity (χ^2^(1) = 11.10, p = 0.001, Cramér’s V = 0.16), and histological grade (χ^2^(1) = 11.50, p = 0.001, Cramér’s V = 0.16).

Age showed a small effect size at the threshold of statistical significance (χ^2^(1) = 3.86, p = 0.050, Cramér’s V = 0.09). No statistically significant differences were found for body mass index (χ^2^(3) = 7.49, p = 0.058, Cramér’s V = 0.13) or molecular subtype (χ^2^(3) = 3.25, p = 0.355, Cramér’s V = 0.08).

Although several variables reached statistical significance, effect size estimates indicated stronger associations for socioeconomic factors (occupation and education), whereas clinical variables showed small to moderate effect sizes (Table 1).

Overall survival at 10 years was 84% (n = 390). According to insurance status, 10-year survival was 95.9% (n = 223) among insured patients compared with 75.9% (n = 176) among uninsured patients (Table 2).

**Table 2:** Kaplan-Meier overall survival at one, three, five, and 10 years by insurance status. Kaplan-Meier estimated overall survival. Percentages represent survival probability at each time point.

Time (Years)	Overall, n (%)	Insured, n (%)	Uninsured, n (%)
1	454 (97.7)	231 (99.6)	223 (95.9)
3	441 (95.0)	229 (98.7)	212 (91.2)
5	415 (89.4)	226 (97.5)	193 (83.1)
10	390 (84.0)	223 (95.9)	176 (75.9)

**Figure 1:**
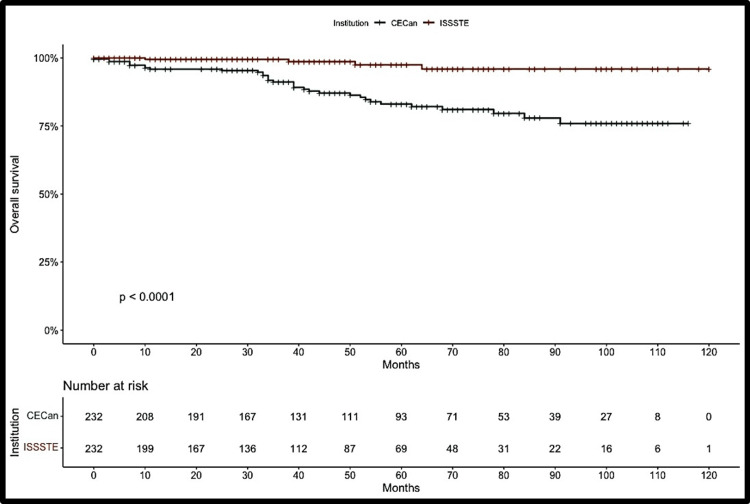
Kaplan-Meier survival curve with log-rank test in breast cancer patients according to health insurance status. CECan: State Cancer Center “Dr. Miguel Dorantes Mesa”; ISSSTE: Institute of Security and Social Services for State Workers

Kaplan-Meier curves showed greater cumulative mortality among uninsured patients during follow-up, with a more pronounced decline between 30 and 40 months after diagnosis. In contrast, survival among insured patients remained relatively stable throughout the 120-month follow-up period. The log-rank test demonstrated a statistically significant difference in survival between groups (p < 0.001; Figure 1).

To evaluate the association between survival and healthcare system type, Cox proportional hazards regression was performed. In the unadjusted analysis, uninsured status was associated with a higher risk of death. This association remained statistically significant after adjustment, with an HR of 4.70 (95% CI: 1.66-13.52) (Table 3).

**Table 3:** Cox proportional hazards models for overall survival according to health insurance status. The adjusted model included age, education level, occupation, area of residence, clinical stage, lymph node involvement, molecular subtype, and treating institution. Outcome: overall survival. Proportional hazards assumptions were evaluated using Schoenfeld residuals. HR: hazard ratio; CI: confidence interval

Variable	Crude HR (95% CI)	Adjusted Model HR (95% CI)	p-value
Health insurance status			
Insured (reference)	1	1	
Uninsured	4.7 (1.35-16.56)	4.7 (1.66-13.52)	0.004

## Discussion

This study compared BC survival and patient characteristics between two Mexican health institutions operating under different insurance schemes. Overall survival was 89.4% at five years and 84.0% at 10 years, consistent with national estimates. Unger-Saldaña et al. reported a five-year survival of 89.0%, highlighting the continued relevance of healthcare access and social conditions in BC outcomes in Mexico [3].

The mean age at diagnosis was 55 ± 12 years, similar to previous national reports (53-55 years) [10,11]. The relatively younger age at diagnosis compared with high-income countries has been consistently described in Latin America and is associated with a greater social and economic burden [5-7]. Younger age has also been identified as a prognostic factor in specific subgroups, particularly among very young women [7].

Comorbid conditions were frequent. The prevalence of diabetes (17.5%) and hypertension (29.1%) exceeded national estimates from the 2021 National Health and Nutrition Survey [12]. In addition, nearly four out of five patients were overweight or obese at diagnosis, consistent with previous studies in Mexican populations [12-16]. Excess body weight has been associated with poorer prognosis and increased mortality, supporting the clinical relevance of these findings.

Educational attainment emerged as an important differentiating factor. More than half of the patients had only basic education, a condition associated with lower participation in screening, delayed healthcare seeking, and presentation with advanced-stage disease [17,18]. In contrast, higher educational levels have been linked to greater health awareness and earlier use of health services, underscoring education as a key social determinant of BC outcomes.

From a pathological perspective, the predominance of ductal and lobular carcinomas and the near-equal laterality distribution were consistent with national and international evidence [10,15,19], suggesting that tumor biology alone is unlikely to explain the survival differences observed between institutions.

Clear sociodemographic differences were identified between insurance groups. Insured patients were more frequently employed, had higher educational attainment, and lived in urban areas. In contrast, uninsured women were more often homemakers, had lower education levels, and resided in rural settings. These patterns reflect the structure of the Mexican health system, in which insurance coverage is linked to formal employment, and are consistent with previous regional studies [20-22].

Clinical stage at diagnosis differed substantially between groups. Insured patients were more often diagnosed at earlier stages, whereas uninsured patients more frequently presented with advanced disease. Advanced stage and unfavorable tumor characteristics are well-established predictors of mortality [23]. In addition, socioeconomic disadvantage, lack of insurance, and limited education have been consistently associated with lower survival across diverse settings [24].

Survival analysis demonstrated marked differences between groups. Five-year Kaplan-Meier survival was 97.5% (n = 229) among insured patients compared with 83.1% (n = 204) among uninsured patients. These differences were observed despite a higher proportion of poorly differentiated tumors among insured women. Although treatment timeliness, adherence, and quality of care were not directly assessed, the observed patterns are consistent with previous studies reporting associations between healthcare access, stage at diagnosis, and survival [25].

Differences in patient case-mix should also be considered. Public oncology centers may receive referrals of patients with more complex or advanced disease, which could introduce referral bias and contribute to the observed survival differences. In addition, a large proportion of the Mexican population relies on state health services, potentially increasing demand and patient volume in these facilities [20]. Although higher hospital volume has generally been associated with improved outcomes due to greater clinical experience and multidisciplinary care [26], the survival differences observed in this study are more likely related to variations in patient characteristics at presentation rather than to institutional performance alone.

In multivariable Cox analysis, care within the public system was associated with a higher hazard of death compared with care in the insured system. This finding is consistent with international evidence indicating that insurance status and sociodemographic conditions are associated with survival differences across healthcare settings [27]. Structural and social inequities, including barriers to preventive services, delayed diagnosis, and differences in continuity of care, have been widely described as contributors to BC outcome disparities in vulnerable populations [28,29].

BC survival in Mexico appears to be shaped by the combined influence of stage at diagnosis, social and demographic factors, and the healthcare system in which treatment is provided. Addressing disparities in early detection and ensuring fair access to diagnostic and therapeutic services are critical steps toward improving outcomes across the population.

Limitations

This study has several limitations. Its retrospective design relied on clinical records not originally intended for research, which may have introduced information bias and incomplete data. The inclusion of patients from only two institutions may limit generalizability and introduce selection bias related to institutional sampling. Differences in patient case-mix between institutions, including possible referral of more severe cases to specialized centers, may also have influenced the observed survival differences.

Lead-time bias cannot be excluded, as earlier diagnosis among insured patients may have contributed to longer observed survival independent of prognosis. Information on treatment modalities, adherence, quality-of-care indicators, and time from diagnosis to treatment initiation was not available. Socioeconomic information was limited to proxy measures (education, occupation, and residence), which may not fully capture social disadvantage and may result in residual confounding. In addition, staging accuracy depended on routine clinical records, and detailed molecular or genetic tumor characteristics were not assessed.

Despite these limitations, the study has important strengths, including a relatively large cohort with long-term follow-up, mortality verification through clinical records, comparison of two healthcare systems within the same geographic context, and the use of standardized survival methods, providing relevant real-world evidence on institutional and sociodemographic disparities.

## Conclusions

This study identified differences in the sociodemographic and clinical profiles of women with BC according to health insurance status and type of healthcare institution in Veracruz. Insured patients were more likely to have higher educational attainment, formal employment, urban residence, and earlier clinical stages at diagnosis, whereas uninsured patients more frequently had lower educational levels, were homemakers, lived in rural areas, and presented with advanced disease. These differences were also associated with lower survival among uninsured patients.

The findings suggest that health insurance status and institutional context are associated with variations in patient characteristics and survival. These results underscore the need to strengthen equity and standardization in care processes across health systems and provide relevant information to inform public health strategies aimed at improving access to care, promoting early diagnosis, and reducing BC mortality.
